# Co-occurring dominance and ideal point processes: A general IRTree framework for multidimensional item responding

**DOI:** 10.3758/s13428-024-02405-4

**Published:** 2024-04-16

**Authors:** Viola Merhof, Thorsten Meiser

**Affiliations:** Department of Psychology, University of Mannheim, L 13 15, D-68161 Mannheim, Germany

**Keywords:** IRTree models, Ideal point models, Dominance models, Multidimensional IRT, Response styles

## Abstract

Responding to rating scale items is a multidimensional process, since not only the substantive trait being measured but also additional personal characteristics can affect the respondents’ category choices. A flexible model class for analyzing such multidimensional responses are IRTree models, in which rating responses are decomposed into a sequence of sub-decisions. Different response processes can be involved in item responding both sequentially across those sub-decisions and as co-occurring processes within sub-decisions. In the previous literature, modeling co-occurring processes has been exclusively limited to dominance models, where higher trait levels are associated with higher expected scores. However, some response processes may rather follow an ideal point rationale, where the expected score depends on the proximity of a person’s trait level to the item’s location. Therefore, we propose a new multidimensional IRT model of co-occurring dominance and ideal point processes (DI-MIRT model) as a flexible framework for parameterizing IRTree sub-decisions with multiple dominance processes, multiple ideal point processes, and combinations of both. The DI-MIRT parameterization opens up new application areas for the IRTree model class and allows the specification of a wide range of theoretical assumptions regarding the cognitive processing of item responding. A simulation study shows that IRTree models with DI-MIRT parameterization provide excellent parameter recovery and accurately reflect co-occurring dominance and ideal point processes. In addition, a clear advantage over traditional IRTree models with purely sequential processes is demonstrated. Two application examples from the field of response style analysis highlight the benefits of the general IRTree framework under real-world conditions.

Likert-type rating scales are widely used to assess personality, attitudes, or beliefs via self-reports, and they are omnipresent in research and applied fields of psychology and social sciences. A popular item response theory (IRT) approach for analyzing such rating data are item response tree (IRTree) models (Böckenholt, 2012; De Boeck & Partchev, 2012; Jeon & De Boeck, 2016; Böckenholt & Meiser, 2017), which have been proven to be a broadly applicable model class offering high flexibility with regard to investigating response processes underlying respondents’ judgments and decisions.

**Fig. 1:**
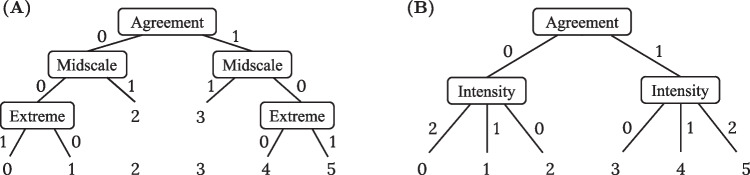
Tree diagrams for decomposing responses to six-point rating items into sub-decisions. **A** Decomposition into three sub-decisions by five binary pseudo-items. Adapted from Böckenholt (2017). **B** Decomposition into two sub-decisions by binary and ordinal (three-step) pseudo-items. Adapted from Meiser et al. (2019)

A key characteristic of IRTree models is their multidimensional nature with the underlying assumption that multiple response processes are sequentially^1^ involved in the selection of response categories. This property arises from the decomposition of the ordinal rating responses into a sequence of pseudo-items, which represent the sub-decisions assumed to be taken by the respondents during response selection. For example, respondents may first decide on whether to agree or disagree with an item, and subsequently make fine-grained decisions among the available agreement or disagreement categories. Such sub-decisions are typically assumed to be binary judgments, though the pseudo-items can likewise be defined as ordinal judgments with three or more options (see Meiser et al., 2019, and Fig. 1). The pseudo-items are modeled by separate IRT models, and by assigning different latent traits to the sub-decision, their effects on the response selection can be disentangled. Thus, IRTree models can capture multidimensional response processes, even though making use of unidimensional IRT modeling for the individual pseudo-items.

A typical aim of using IRTree models is to separate the effects of substantive traits from those of response styles (RS) – individual preferences for specific response categories of rating scales irrespective of item content (for an overview, see Van Vaerenbergh & Thomas, 2013). For instance, some respondents may prefer categories located in the middle of the response scale (midscale response style; MRS), whereas others may rather prefer clear-cut responses and tend to select the extreme categories (extreme response style; ERS). Such different usages of the response scale can systematically distort the estimation of individual substantive trait levels, group means, and correlations among multiple traits, so that RS must be controlled for to obtain valid measurements (Baumgartner & Steenkamp, 2001; Alwin, 2007). Commonly used IRTree models for the analysis of RS define agreement decisions as dependent on the substantive trait levels of respondents, whereas more fine-grained decisions are modeled to be based on individual RS, like the judgment to give extreme versus non-extreme responses guided by ERS, or the judgment to select the neutral middle category guided by MRS (e.g., Böckenholt, 2017; Khorramdel & von Davier, 2014; Plieninger & Meiser, 2014; Thissen-Roe & Thissen, 2013). However, even though IRTree models are mostly used for RS analysis, they are flexible to incorporate any kind of person-specific influences on the selection of individual response categories (e.g., socially desirable responding) by defining the pseudo-items correspondingly.

In contrast to this flexibility of IRTree models with regard to including various latent traits in the pseudo-items, little attention has so far been devoted to their flexibility in terms of modeling both monotonous and non-monotonous effects of such traits on the response selection. This property is described by the item response function (IRF), which defines how each value of the latent trait continuum maps to the expected score of an item. For ordinal item responses $Y \in \{0,...,K\}$ on a rating scale with *K*+1 categories, the IRF of trait $\theta$ is given by

1 $$\begin{aligned} \text {IRF}(\theta ) = \sum _{y=0}^K y \cdot p(Y=y\,|\,\theta ). \end{aligned}$$

Thus, the IRF defines the expected value of *Y* for a given trait level $\theta$ depending on the category-specific probabilities that are specified by the IRT model (e.g., the generalized partial credit model; GPCM; Muraki, 1992).

**Fig. 2:**
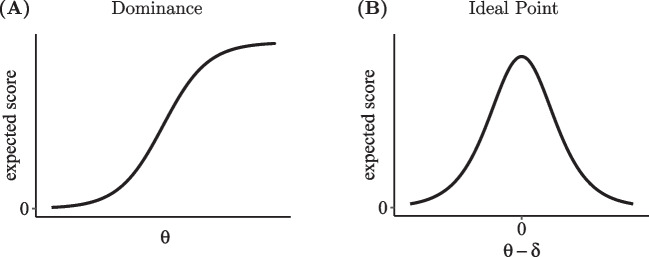
Item response functions under the dominance and ideal point assumption

IRT models can be grouped into two classes based on their IRFs: Dominance and ideal point models (Coombs, 1964). They go back to Likert (1932) and Thurstone (1928), respectively, and both have a long history in the psychometric literature. Typically, IRTree decision processes are assumed to follow the dominance rationale, meaning that a higher trait level is modeled to result in a higher expected score of the respective pseudo-item (see Fig. 2A). As suggested by the term *dominance*, respondents overcome an item if their trait level values exceed the item’s level of difficulty. For instance, the probability to agree with an item, which states that environmental protection is an important issue, increases with higher levels of environmental awareness of the respondents. Dominance models have monotonically increasing IRFs and frequently applied members of this class are the models of the Rasch family; for example, the Rasch model (Rasch, 1960) or 2PL model (Birnbaum, 1968) for binary items, or the GPCM for ordinal items. An alternative assumption is captured by *ideal point* models, in which the relationship between the expected score and the latent trait is unimodal and non-monotonic (see Fig. 2B). The expected score is highest if a respondent’s trait level, which is called their ideal point, matches the item’s location, and decreases with larger distances. The more the trait levels deviate from the item location in an upward or downward manner, the stronger respondents will dismiss the item content from above or from below, respectively. For example, respondents with moderate environmental awareness may agree with the statement that the current environmental regulations are adequate, whereas respondents who prefer either stricter or less strict regulations disagree, though for different reasons. As the IRFs are symmetrical about the item location, only the proximity of a respondent and the item, not the direction of a deviation, is relevant for the response selection. Several IRT models for dichotomous and polytomous items have been developed for the ideal point rationale, like the hyperbolic cosine model (Andrich & Luo, 1993; Andrich, 1995) or the generalized graded unfolding model (Roberts et al., 2000).

Although rating scale items are mostly constructed under and analyzed by dominance approaches, there is compelling evidence that ideal point models often better describe item responding of non-cognitive constructs, and thus, should be considered when analyzing self-reported data (e.g., Liu & Wang, 2016; Roberts & Laughlin, 1996; van Schuur & Kiers, 1994; for an overview, see Drasgow et al., 2010). Nevertheless, compared to dominance IRT modeling, there is little research on multidimensional response processes so far, and most ideal point models treat item responses as solely dependent on the substantive trait to be measured, while ignoring possible other influences. This poses a threat to the validity of ideal point models whenever RS or other additional response processes are involved in item responding (Liu & Wang, 2019).

Multidimensional processes should, therefore, be investigated not only for dominance but also for ideal point items. However, this can require integrating response processes with different IRFs into multidimensional models, for instance, if RS are to be considered. Such are by definition dominance processes since higher RS levels reflect stronger preferences for certain response categories. A straightforward way to include RS into the analysis of ideal point items are IRTree models, as the pseudo-items are parameterized independently of each other, and thus, processes of different IRFs can be defined by existing unidimensional IRT models of the dominance and ideal point rationale. Indeed, Jin et al. (2022) demonstrated the advantages of such an IRTree model, in which an ideal point model was applied to a trait-based sub-decision and a dominance model to an ERS-based sub-decision. In two application examples of attitudinal questionnaires, the authors showed that their model fit the data better than both an ideal point model ignoring RS, and classical dominance IRTree models accounting for RS.

This example nicely illustrates that the decomposition of multidimensional item responses into unidimensional decision processes with different kinds of IRFs provides high flexibility while keeping the modeling complexity low. Still, this advantage comes at the cost of the simplistic assumption that each cognitive processing step during response selection is based on one response process at a time (e.g., either the substantive trait or a RS). However, multiple response processes may not only contribute to item responding sequentially, but may also occur simultaneously on the level of sub-decisions. Such co-occurring processes can likewise be integrated into IRTree models by replacing the traditionally used unidimensional pseudo-items with multidimensional IRT (MIRT) models (von Davier & Khorramdel, 2013; see Jeon & De Boeck, 2016; Meiser et al., 2019). In RS analysis, for example, Meiser et al. (2019) showed that the selection of more or less extreme response categories was not only dependent on the individual ERS, but further influenced by the substantive trait levels of respondents. Such multidimensional parameterizations of pseudo-items allow the investigation of more complex and presumably more realistic hypotheses about the cognitive processing during item responding, and they were shown to be preferable over unidimensional ones with regard to psychometric properties (Merhof & Meiser, 2023).

Nevertheless, multidimensional pseudo-items have so far been exclusively applied to combinations of dominance processes. Dominance MIRT models can be derived from unidimensional ones by extending a single latent trait to a linear combination of multiple traits, and thus reflect the assumption that several processes contribute to the response selection in a cumulative, additive way (e.g., Bolt & Johnson, 2009; Bolt & Newton, 2011; Falk & Cai, 2016; Henninger & Meiser, 2020; Jin & Wang, 2014). Ideal point processes, in contrast, must not be considered additive to other processes, as this would counteract the proximity concept.^2^ Therefore, modeling co-occurring response processes under the ideal point assumption is less straightforward and there exist only few models that address this challenge, all of which focus on modeling RS in addition to trait-based responding to ideal point items. For instance, the approaches by Luo (1998); Wang et al. (2013) implicitly account for RS in ideal point items by defining random category thresholds which vary across persons. Javaras and Ripley (2007) and Liu and Wang (2019) also use random thresholds, though specify such explicitly as a linear combination of different RS. However, none of the models treats RS as independent, stand-alone response processes, but all rather assume that they can only occur in the presence of another trait-based process, as they are defined as person-specific shifts of trait-based responding. In contrast, our understanding of item responding in the framework of IRTree models is that trait-based and RS-based responding are distinct processes, which can make both individual and combined contributions to the sub-decisions during response selection. Further, the models are only adapted to the co-occurrence of an ideal point trait and dominance RS, and do not generalize to other types of response processes with any IRF. None of the models provides a general formulation that consistently connects multiple response processes independent of dominance and ideal point assumptions.

Therefore, the aim of the present article is to provide a general IRTree framework which is independent of the choice of dominance or ideal point modeling, and in which multiple response processes can be involved in the response selection (a) sequentially across pseudo-items, and (b) as co-occurring processes within pseudo-items. While sequential multidimensionality can be implemented using existing IRT modeling, we propose a new approach for co-occurring response processes, in which multiple dominance processes, multiple ideal point processes, as well as a combination of both are modeled in a consistent manner. The new MIRT model of co-occurring dominance and ideal point processes (DI-MIRT) is based on the divide-by-total framework for ordinal item responses (see Thissen & Steinberg, 1986). It can be used independently of IRTree models, though in this article we focus on that model class and demonstrate that a DI-MIRT parameterization can specifically benefit IRTree pseudo-items: The new DI-MIRT model can not only be used for modeling multidimensional response processes in ideal point items (see section “Response style analysis in idealpoint items”), but also for including ideal point processes into dominance items (e.g., when modeling the selection of midscale response categories; see section “Middle categoriesin dominance items”).

Furthermore, this article highlights the flexibility of the proposed general IRTree framework, which can be considered a modular system with three independent components that can be combined as desired. One component of IRTree models is the psychological theory concerning the decomposition of ordinal rating responses into sub-decisions. By specifying the number and structure of the sub-decisions, theory-driven hypotheses on the logical sequence of cognitive processing stages can be defined (see Fig. 1). A second component is the definition of the response processes that contribute to the individual sub-decisions. Since various processes can be assigned to the pseudo-items separately from each other, personal characteristics may be involved in one or more pseudo-items, and pseudo-items may depend on one or more processes. Using the new DI-MIRT model, it is now possible to define a third IRTree component independently of the other two, which is the choice of process-specific IRFs. The individual response processes can be parameterized by dominance or ideal point models, and they can be freely combined both within and between pseudo-items.

In the remainder of this article, we will illustrate this modular system and present exemplary IRTree models that differ in terms of the selection and combination of the three components. To this end, we firstly derive the new DI-MIRT model of co-occurring processes from existing models of the divide-by-total framework. Secondly, we introduce IRTree models in which dominance and ideal point processes co-occur within sub-decisions. Thirdly, a simulation study on parameter recovery and model selection is presented. Then, the utility of the new approach is illustrated by two empirical examples, the first one focusing on the investigation of the relative importance of co-occurring processes in IRTree sub-decisions, and the second one using response time data to provide a construct validation of the parameter estimates. We conclude with a discussion of the results.

**Fig. 3:**
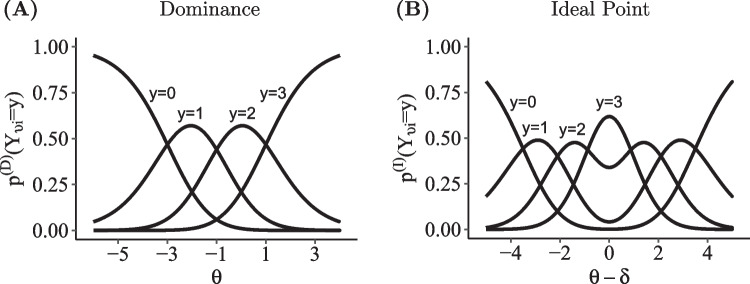
Category Probability curves for responding to four-point rating items. Category probabilities under the dominance assumption of the GPCM (**A**) and under the ideal point assumption of the GGUM (**B**). Discrimination parameters $\alpha$ and $\lambda$ are set to 1; the thresholds are set as follows: $\beta _1=-3$; $\beta _2=-1$; $\beta _3=1$; $\xi _1=-3.5$; $\xi _2=-2.1$; $\xi _3=-0.6$

## Existing dominance and ideal point divide-by-total models

The divide-by-total framework contains both dominance and ideal point models. For both of them holds that the category probabilities of ordinal responses $Y \in \{0,...,K\}$ are defined as the ratio of category-specific components divided by the sum of the $K+1$ components of all available categories, so that the probabilities across categories sum up to 1. For modeling item responses of person $v = 1,...,N$ to item $i = 1,...,I$ under the dominance assumption (D), divide-by-total models can take the form

2 $$\begin{aligned} p^{(D)}(Y_{vi} = y_{vi}) = \frac{\omega _{viy}}{\sum \limits _{j=0}^{K}\omega _{vij}} = \frac{\text {exp}(\eta _{viy})}{\sum \limits _{j=0}^{K}\text {exp}(\eta _{vij})}, \end{aligned}$$

where $\eta _{viy}$ is a linear combination of person and item parameters.

A prominent member of dominance divide-by-total models is the generalized partial credit model (GPCM; Muraki, 1992), which is given by

3 $$\begin{aligned} p^{(D)}(Y_{vi} = y_{vi} \, | \,\varvec{s}, \varvec{\theta }, \varvec{\alpha }, \varvec{\beta }) = \frac{\text {exp}\left[ \alpha _i \left( s_y \theta _v - \sum \limits _{k=0}^y \beta _{ik} \right) \right] }{\sum \limits _{j=0}^{K}\text {exp}\left[ \alpha _i \left( s_j\theta _v - \sum \limits _{k=0}^j \beta _{ik} \right) \right] }, \end{aligned}$$

with $\beta _{i0} := 0$ and $s_y = y$. $\theta _v$ denotes the person-specific trait level, $\alpha _i$ the item-specific discrimination parameter, and $\beta _{ik}$ the item- and category-specific thresholds (or difficulties). The threshold parameters can be rewritten as $\beta _{ik} =\beta _i + \zeta _{ik}$, where $\beta _i$ denotes the item location and is defined as $\sum _{k=1}^K \beta _{ik}/K$ and $\zeta _{ik}$ denotes the category-specific deviations. If the thresholds $\beta _{ik}$ are ordered across the ordinal categories, each category has a section on the latent trait continuum for which the probability to be chosen is higher than the probabilities of all other categories. The scoring weights $\varvec{s}$ define the relation between trait and response categories and are fixed to $s_y = y$ in the GPCM, reflecting that the response categories are ordered and that higher trait levels are associated with higher categories. However, they can be set to any other values depending on the theoretical assumptions, or can be estimated like in the nominal response model for categorical responses (Bock, 1972; Thissen et al., 2010). Note that the choice of scoring weights depends on the assumption of how the trait influences the selection of categories, but does not change the underlying dominance assumption. Figure 3A shows exemplary category probability curves for an item on a four-point scale under the GPCM.

For modeling item responses under the ideal point assumption (I), divide-by-total models can take the form

4 $$\begin{aligned} p^{(I)}(Y_{vi} = y_{vi}) = \frac{\omega _{viy}}{\sum \limits _{j=0}^{K}\omega _{vij}} = \frac{ \text {exp}(\eta _{1viy}) + \text {exp}(\eta _{2viy}) }{ \sum \limits _{j=0}^{K}\big (\text {exp}(\eta _{1vij}) +\text {exp}(\eta _{2vij}) \big ) }, \end{aligned}$$

where $\eta _{1viy}$ and $\eta _{2viy}$ are linear combinations of person and item parameters. The category-specific components $\omega _{viy}$ are defined as the sum of two exponential terms since it is assumed that each response category of a rating scale is composed of two unobservable, latent categories. Each of the two associated latent categories reflects the perspectives from above and from below the item location, respectively. For instance, the observable categories “agree” and “disagree” of a dichotomous item correspond to the latent categories “disagree from below”, “agree from below”, “agree from above”, and “disagree from above”. Thus, ideal point divide-by-total models account for two different reasons for which respondents can select a specific category, such as disagreement being chosen because of having a much higher or a much lower ideal point compared to the item location. By adding up the probabilities of selecting a category from below and from above, probabilities of the observable categories are obtained.

The generalized graded unfolding model (GGUM; Roberts et al., 2000) belongs to the class of ideal point divide-by-total models and is given by

5 $$\begin{aligned}&p^{(I)}(Y_{vi} = y_{vi} \, | \, \varvec{s}, \varvec{\theta }, \varvec{\lambda }, \varvec{\delta }, \varvec{\xi } ) = \nonumber \\&\frac{ \text {exp}\left[ \lambda _i \left( s_y (\theta _v-\delta _i) - \sum \limits _{k=0}^y \xi _{ik} \right) \right] + \text {exp}\left[ \lambda _i \left( (M-s_y) (\theta _v-\delta _i) - \sum \limits _{k=0}^y \xi _{ik} \right) \right] }{ \sum \limits _{j=0}^{K}\left\{ \text {exp}\left[ \lambda _i \left( s_j(\theta _v-\delta _i) - \sum \limits _{k=0}^j \xi _{ik} \right) \right] + \text {exp}\left[ \lambda _i \left( (M-s_j)(\theta _v-\delta _i) - \sum \limits _{k=0}^j \xi _{ik} \right) \right] \right\} } \end{aligned}$$

with $\xi _{i0} := 0$, $M=2K+1$ and $s_y=y$. $\theta _v$ denotes the person’s trait level (ideal point), $\delta _i$ the item location, $\lambda _i$ the discrimination parameter, and $\xi _{ik}$ the category-specific threshold. If the thresholds $\xi _{ik}$ are $<0$ and ordered across the ordinal categories, all observable categories have sections on the latent trait continuum for which the probability to be chosen is higher than for the other categories. Note that the parameterization of each exponential term has high similarity to the GPCM, which in fact causes the category probability curves of the $M+1$ latent categories to take the form of a GPCM. The first exponential term in the numerator and denominator of the GGUM corresponds to latent response categories from below, whereas the second term corresponds to latent categories from above. Each two associated latent categories differ only in their scoring weights of the person-item proximity $(\theta _v-\delta _i)$, which are defined as *y* and $(M-y)$, respectively. Thus, the scoring weights of latent categories from below increase across categories (0, ..., *K*), whereas they decrease to the same extent for latent categories from above $(M,...,K+1)$. The observable category probabilities are symmetrical about the point $(\theta _v - \delta _i) = 0$, which implies that selecting response category *y* is equally likely for a positive or negative deviation of a respondent’s trait level from the item location. As is the case for dominance models, the scoring weights of ideal point divide-by-total models can be fixed to any values, which would reflect different hypotheses on the relation between trait and categories, without changing the ideal point rationale. Figure 3B shows exemplary category probability curves for an item on a four-point scale under the GGUM.

**Fig. 4:**
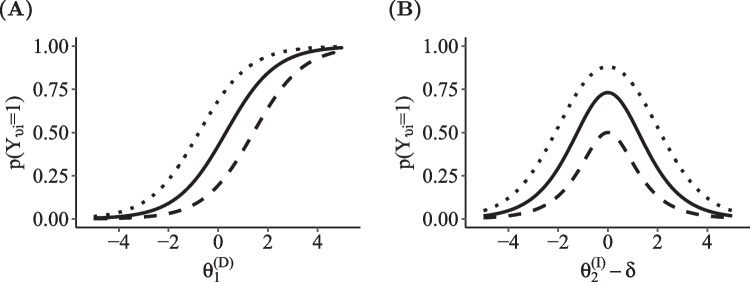
Probability curves for endorsing a binary item under the DI-MIRT model. One response process follows the dominance assumption parameterized by the GPCM ($\theta _1^{(D)}$) and the other process follows the ideal point assumption parameterized by the GGUM ($\theta _2^{(I)}$). **A** Probability curves for fixed $|\theta _2^{(I)}-\delta |$ of .5 (*dotted line*), 1.5 (*solid line*), and 2.5 (*dashed line*). **B** Probability curves for fixed $\theta _1^{(D)}$ of 1 (*dotted line*), 0 (*solid line*), and -1 (*dashed line*). The other parameters are set as follows: $\alpha = 1$, $\lambda = 1$, $\beta _1 = 0$, $\xi _1 = -1$, $\varvec{s}^{(D)} = (0,1)$, and $\varvec{s}^{(I)} = (0,1)$

## Co-occurring dominance and ideal point processes

The novel DI-MIRT model of co-occurring processes is a multi-process generalization of dominance and ideal point divide-by-total models and includes both of them as special cases. In order to combine response processes described by those two models, the definitions of dominance and ideal point approaches must be brought into the same format. As described above, ideal point divide-by-total models consist of the sum of two exponential terms, which correspond to the two underlying latent categories together defining the probability distribution of observable categories. For dominance models, in contrast, the probability distribution of observable categories can be modeled directly. Nonetheless, such models can likewise be displayed in the form of two added components, by applying the single linear parameter combination of a dominance model (which is $\eta _{viy}$ in Eq. 2) to both exponential terms in the ideal point formulation ($\eta _{1viy}$ and $\eta _{2viy}$ in Eq. 4). Consequently, all category-specific components $\omega _{viy}$ are simply doubled both in the numerator and denominator, which does not affect the probability distribution across categories, so an equivalent model results. Metaphorically speaking, each observable response category is artificially divided into two latent categories, which are selected with equal probability. Thereby, ideal point and dominance models can be expressed in the same form and are represented by two added components. If several response processes $r \in \{1,...,R\}$, no matter if dominance or ideal point processes, are to be aggregated to a common probability distribution, the respective linear parameter combinations can simply be added within each of the two exponential terms. The resulting DI-MIRT model is given by

6 $$\begin{aligned} p(Y_{vi} = y_{vi}) = \frac{ \text {exp}\left( \sum \limits _{r=1}^{R}\eta _{1viyr}\right) + \text {exp} \left( \sum \limits _{r=1}^{R}\eta _{2viyr}\right) }{ \sum \limits _{j=0}^{K}\left[ \text {exp}\left( \sum \limits _{r=1}^{R}\eta _{1vijr}\right) + \text {exp} \left( \sum \limits _{r=1}^{R}\eta _{2vijr}\right) \right] }. \end{aligned}$$

With this general formulation, the co-occurrence of several dominance processes, several ideal point processes, or a combination of both can be modeled in a consistent way. Note that the two linear parameter combinations do not differ for dominance response processes ($\eta _{1vijr} = \eta _{2vijr}$), whereas the parameterizations for ideal point processes differ in their scoring weights (see Eq. 5). In the further course of the article, we use the GPCM for modeling dominance processes and the GGUM for ideal point processes. However, the individual processes can be defined by any unidimensional dominance or ideal point IRT model which can be represented in the form of divide-by-total models (as defined in Eqs. 2 and 4). Figure 4 illustrates the co-occurrence of one dominance and one ideal point process for an exemplary binary item. The higher a person’s dominance trait level ($\theta _1^{(D)}$) and the higher the proximity of a person’s ideal point to the item location ($|\theta _2^{(I)}-\delta |$), the higher the probability of endorsing the item.

### Identification

The DI-MIRT model is a suitable theoretical model of how co-occurring processes jointly determine item responding, though it is highly parameterized and not identified without certain constraints, whenever two or more processes are to be considered. Given that the linear parameter combinations of the co-occurring processes ($\eta _{1viyr}$ and $\eta _{2viyr}$) each consist of a person-specific trait and item-specific category thresholds, some restriction may arise with respect to estimating those two kinds of parameters.

Firstly, the threshold parameters of several processes cannot be separated, so only one common threshold per category can be estimated. This common threshold, which we call *category intercept*, is a weighted linear combination of the threshold parameters of the co-occurring processes. It is, therefore, not possible to examine the individual contributions of the involved processes to the size of each category intercept, that is, to why specific response categories are selected more or less frequently. For instance, for dominance processes modeled by the GPCM and ideal point processes modeled by the GGUM, the linear parameter combinations are defined as

7 $$\begin{aligned} \sum \limits _{r=1}^{R}\eta _{1viyr}= & {} \sum _{r=1}^{R} \Bigg [ (\alpha _{ir} s_{yr}\theta _{vr} )^{m_r}\nonumber \\{} & {} + \big (\lambda _{ir} s_{yr} (\theta _{vr} - \delta _{ir}) \big )^{(1-m_r)} \Bigg ] - \sum \limits _{k=0}^y \tau _{ik} \end{aligned}$$

and

8 $$\begin{aligned} \sum \limits _{r=1}^{R}\eta _{2viyr}= & {} \sum _{r=1}^{R} \Bigg [ (\alpha _{ir} s_{yr}\theta _{vr} )^{m_r} + \big (\lambda _{ir} (M-s_{yr})\nonumber \\{} & {} (\theta _{vr} - \delta _{ir}) \big )^{(1-m_r)} \Bigg ] - \sum \limits _{k=0}^y \tau _{ik}, \end{aligned}$$

where $m_r=1$ if process *r* is a dominance process and $m_r=0$ if it is an ideal point process. $\tau _{ik}$ is the category intercept of the *R* processes and is given by

9 $$\begin{aligned} \tau _{ik}=\sum _{r=1}^{R} (\alpha _{ir} \beta _{ikr})^{m_r} + (\lambda _{ir} \xi _{ikr})^{(1-m_r)}, \end{aligned}$$

where $\tau _{i0} = 0$ as a consequence of the previous definitions. Note that this constraint of only the common category intercept of several processes being estimated applies to existing MIRT models as well (such as the multidimensional nominal response model; Bolt & Johnson, 2009), though it is implicitly captured in the model formulations by defining only one threshold in the first place.

The second constraint of the DI-MIRT model is that the respondents’ trait levels can only be separated from each other if the scoring weights differ in at least one of the two exponential terms. Therefore, two (or more) dominance processes or two (or more) ideal point processes must be defined to affect the response categories in different ways. Again, this also applies to other MIRT models. For instance, in multidimensional models for the analysis of ERS, the ERS-based process typically gets assigned the scoring weights (1, 0, ..., 0, 1), reflecting the assumption that only the outermost two categories are affected, and thus can be separated from the ordinal influence of a trait with scoring weights (0, ..., *K*).

In addition to the above remarks, it should be noted that the scale of the latent continuum is not per se identified in the DI-MIRT model – as is the case for any other IRT model. When estimating the DI-MIRT model, the location, the variability, and the orientation of the continuum have to be fixed. The identification of the location is required in all IRT models and is typically done by setting the mean of the latent trait distribution to zero. Models with discrimination parameters, such as the GPCM, additionally require fixing the variability, which is often done by setting the variance of the trait distribution to one. In ideal point models, such as the GGUM, the orientation (or sign) of the continuum is unknown and therefore has to be fixed. The non-identified orientation is due to the fact that the estimation of the trait levels and item locations in ideal point models is based on their proximity, that is, the pairwise distances on the common latent continuum. Thus, two sets of parameters result in the same likelihood, whereby the two parameter solutions only differ in the signs of the person-specific trait levels and item-specific locations. Importantly, both solutions are equally correct; only the meaning of the latent continuum changes, so it is up to the researcher to decide which of the two parameter sets corresponds to the interpretation of the latent continuum that is more intuitive. The practical specification of the continuum orientation is described below in the context of the simulation study (see “Estimation and Analysis”).

### Probability-based formulation

The DI-MIRT formulation as divide-by-total model given by Eq. 6 can be rewritten as a model which aggregates processes at the level of process-specific category probabilities. Let $\varvec{p}^{(r)}(Y_{vi} = y_{vi})$ denote the vector of probabilities for responding to each of the $K+1$ categories of an item for response process *r*. The joint probability for *R* co-occurring processes can then be obtained by passing the process-specific probabilities to a probability-aggregating function $\sigma$, which is defined as

10 $$\begin{aligned} p(Y_{vi} = y_{vi})&= \sigma _y \left[ \varvec{p}^{(1)}(Y_{vi} = y_{vi}), ...,\varvec{p}^{(R)}(Y_{vi} = y_{vi}) \right] \nonumber \\&= \text {softmax}\left[ \sum \limits _{r=1}^R \text {log}\left( \varvec{p}^{(r)}(Y_{vi}=y_{vi})\right) \right] _{(y+1)}, \end{aligned}$$

with softmax being a normalized exponential function transforming a *Z*-dimensional vector $\varvec{x}$ into a vector of probabilities summing up to 1. Position *z* of this probability vector is given by

11 $$\begin{aligned} \text {softmax} [\varvec{x}]_z = \frac{\text {exp}(x_z)}{\sum \limits _{w=1}^Z\text {exp}(x_w)} \quad \text {for } z = 1,...,Z. \end{aligned}$$

This formulation of the DI-MIRT model with aggregation of response processes on the level of category probabilities is equivalent to the aggregation on the level of linear parameter combinations derived above. Thus, even though these two formulations seem to differ regarding their theoretical assumptions (the aggregation at the level of linear parameter combinations suggests that multiple response processes are simultaneously active; the probability-based approach suggests separate cognitive processing and subsequent aggregation), they cannot be distinguished statistically.

Note, however, that the probability-based model is more general and not restricted to divide-by-total models, since the category-specific probabilities of a process could result from any kind of IRT model (e.g., difference models like the graded response model; Samejima, 1969). Nonetheless, depending on the choice of process-specific models, the estimated parameters might have unintuitive interpretations (e.g., the threshold parameters in a co-occurring model including one process defined by a difference model and another process defined by a divide-by-total model) and different constraints might be necessary in order to identify the models. In this article, we will therefore only refer to processes defined by divide-by-total models, for which both formulations are equivalent.

## IRTree models of co-occurring processes

IRTree models allow to separate the influences of multiple latent traits, which can be involved in the response selection both sequentially across sub-decisions and as co-occurring processes within sub-decisions. The modular system of IRTree models offers high flexibility for the specification of theoretical assumptions with respect to three components: (a) the decomposition of ordinal response into sub-decisions, (b) the response processes involved in such sub-decisions, and (c) the IRFs of the individual processes of each pseudo-item. In this article, we introduce two exemplary IRTree models from the field of RS modeling in which these three components are combined in different ways, and in which dominance and ideal point processes co-occur in at least one pseudo-item. The first model addresses the influences of RS on responding to ideal point items; the second one addresses the selection of middle response categories in dominance items. The two models are formally outlined in the following two sections (“Response style analysis in idealpoint items” and “Middle categories in dominance items”, respectively) and applied to empirical data sets in the section “Empirical Applications”. Even though these exemplary models cover only a part of all conceivable IRTree configurations that arise from the DI-MIRT framework, they illustrate the advantages of the new parameterization for IRTree pseudo-items. Our proposed approach is a rather general one and can be easily adapted to differently structured trees and response processes outside RS modeling. Further, we will only refer to pseudo-items with a maximum of two response processes, although the new approach would allow modeling any number of co-occurring response processes. Within IRTree models, however, we consider this as a reasonable restriction since different processes, like MRS and ERS, are assumed to be sequentially involved in item responding and do not affect the same sub-decisions.

### Response style analysis in ideal point items

**Fig. 5:**
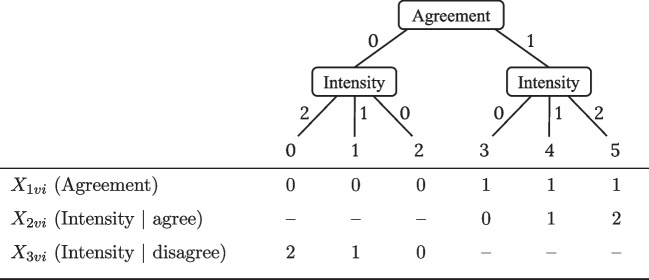
Tree diagram and definition of pseudo-items for responses to six-point rating items. Pseudo-items that are missing by design are marked with ’–’

The first application concerns IRTree models for the analysis of ideal point items while controlling for RS. Assuming that some sub-decisions within the response selection depend on both the trait (i.e., an ideal point process) and a RS (i.e., a dominance process), the model of co-occurring processes is necessary for modeling the respective pseudo-items. We illustrate the analysis of this kind of item response data by an IRTree model of six-point rating scale items, which are decomposed into two sub-decisions of agreement and intensity, as depicted in Fig. 5. The probability of an ordinal response $Y_{vi} \in \{0,...,5\}$ of person $v = 1,...,N$ to item $i = 1,...,I$ is the product of the probabilities of responses to the pseudo-items $X_{hvi}$, where one pseudo-item reflects an agreement decision $(h=1)$ and two pseudo-items reflect the decisions regarding the intensity of responses conditional on the agreement judgment $(h=2 \text { and } h= 3)$:

12 $$\begin{aligned} p(Y_{vi} = y_{vi})= & {} p(X_{1vi} = x_{1vi}) \times p(X_{2vi} =x_{2vi})^{x_{1vi}}\nonumber \\{} & {} \times p(X_{3vi} =x_{3vi})^{(1-x_{1vi})}. \end{aligned}$$

Under the assumption that all sub-decisions are dependent on the substantive trait $\theta$, and that the intensity judgments are additionally affected by the ERS $\eta$, the probabilities of the three pseudo-items can be specified as follows:

13 $$\begin{aligned} p(X_{1vi} = x_{1vi})&= \hspace{21.05501pt}p^{(I)}\Bigl (x_{1vi}\,|\, \varvec{s} = (0,1), \theta _{v}, \lambda _{1i}, \delta _i, \xi _{1i} \Bigr ) \nonumber \\ p(X_{2vi} = x_{2vi})&= \sigma _x\left[ p^{(D)}\Bigl (x_{2vi}\,|\, \varvec{s} = (0,1,2), \eta _{v}, \alpha _{i}, \varvec{\beta }_{1i}\Bigr ),p^{(I)}\Bigl (x_{2vi}\,|\, \varvec{s} = (0,1,2), \theta _{v}, \lambda _{2i}, \delta _i, \varvec{\xi }_{2i}\Bigr ) \right] \nonumber \\ p(X_{3vi} = x_{3vi})&= \sigma _x\left[ p^{(D)}\Bigl (x_{3vi}\,|\, \varvec{s} = (0,1,2), \eta _{v}, \alpha _{i}, \varvec{\beta }_{2i}\Bigr ),p^{(I)}\Bigl (x_{3vi}\,|\, \varvec{s} = (2,1,0), \theta _{v}, \lambda _{2i}, \delta _i, \varvec{\xi }_{3i}\Bigr ) \right] , \end{aligned}$$

where $p^{(D)}$ denotes response probabilities under the GPCM as given in Eq. 3, $p^{(I)}$ denotes probabilities under the GGUM as given in Eq. 5, and $\sigma$ denotes the probability-aggregating function given in Eq. 10. As the sub-decision of agreement depends on a trait-based ideal point process and the intensity decision comprises co-occurring trait and ERS processes, this IRTree structure is abbreviated as $\text {I}_\theta$–$\text {D}_\eta \text {I}_\theta$ in the following.

The scoring weights $\varvec{s}$ of the trait-based ideal point process differ between the two intensity pseudo-items to account for the fact that high proximity of a respondent’s ideal point and the item’s location (i.e., a small difference of $\theta _v$ and $\delta _i$) increases the probability of intense agreement but reduces the probability of intense disagreement. As such intensity scoring weights relate to the definition of the pseudo-items in Eq. 5 from inner to outer ordinal categories of the scale, the respective first weights refer to the least intense ordinal categories (3 and 2 for agreement and disagreement, respectively), followed by the weights for moderately intense (4 and 1) and the most intense (5 and 0) categories. Further, the ordinal definition of the ERS-based dominance process implies that a preference for the extreme categories and a preference for the midscale categories are opposite poles of a common trait. Thus, positive ERS levels increase the probability to select extreme categories and decrease the probability of midscale categories, while it is the other way around for negative ERS levels. Also note that the thresholds of the co-occurring processes within the intensity pseudo-items cannot be separated, meaning that only one category intercept can be estimated. Such category intercepts $\tau$ are defined as given in Eq. 9: For pseudo-item $X_{1vi}$, only one response process is defined, so that the intercept $\tau _{1i}$ is simply given by $\lambda _{1i}\xi _{1i}$. For pseudo-item $X_{2vi}$, the intercept is $\varvec{\tau }_{2i}=\alpha _{i}\varvec{\beta }_{1i} + \lambda _{2i}\varvec{\xi }_{2i}$, and for pseudo-item $X_{3vi}$, it is $\varvec{\tau }_{3i} = \alpha _{i}\varvec{\beta }_{2i} + \lambda _{2i}\varvec{\xi }_{3i}$.

**Fig. 6:**
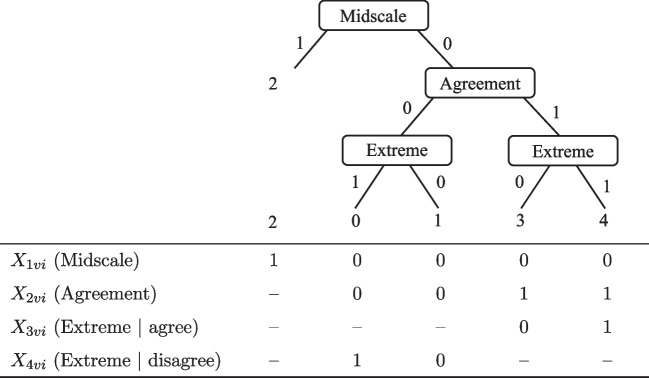
Tree diagram and definition of pseudo-items for responses to five-point rating items. Pseudo-items that are missing by design are marked with ’–’

### Middle categories in dominance items

The second application relates to IRTree sub-decisions of midscale versus non-midscale responding in dominance items. There is an ongoing discussion in the literature on whether middle categories are used as part of the ordinal scale and reflect a neutral attitude of respondents, or whether they are rather considered as a non-response option and selected toavoid providing personal information (e.g., Kalton et al., 1980; Nowlis et al., 2002; Sturgis et al., 2014; Tijmstra & Bolsinova, in press; Tijmstra et al., 2018). The first interpretation implies a trait-based response selection; the latter one indicates that such decisions are based on another trait, which could be referred to as MRS. Thus, it seems reasonable to consider both kinds of response processes in a co-occurring model in order to examine their relative importance for the selection of middle categories.

For such sub-decisions of midscale responding, the substantive trait behaves like an ideal point process, despite the fact the item generally follows the dominance rationale. Therefore, trait-based agreement is modeled as a dominance process, whereas trait-based midscale responding as an ideal point process. This unintuitive property is due to the fact that only respondents who have moderately high substantive trait levels in relation to the item location are assumed to select middle categories as an expression of a neutral opinion. In contrast, respondents having a very high or very low trait level are unlikely to select neutral response categories, because they are assumed to have a clear-cut opinion regarding the item content. Accordingly, if such a trait-based ideal point process is to be modeled to co-occur with a MRS-based dominance process, the DI-MIRT model is required.

This use case of the DI-MIRT model is illustrated by an IRTree model of items on a five-point rating scale with the three sub-decisions of midscale responding, agreement, and extreme responding, as depicted in Fig. 6. The probability of an ordinal response $Y_{vi} \in \{0,...,4\}$ is the product of the conditional pseudo-item probabilities $X_{hvi}$ and is given by:

14 $$\begin{aligned} p(Y_{vi} = y_{vi})= & {} p(X_{1vi} = x_{1vi}) \times \Bigg [\,p(X_{2vi} = x_{2vi})\nonumber \\{} & {} \times p(X_{3vi} = x_{3vi})^{x_{2vi}}\nonumber \\{} & {} \times p(X_{4vi} =x_{4vi})^{(1-x_{2vi})}\Bigg ]^{(1-x_{1vi})}, \end{aligned}$$

Assuming that the decision of midscale responding depends on the substantive trait $\theta$ and the MRS $\eta _1$, that agreement is solely trait-based, and that extreme responding depends on the trait and the ERS $\eta _2$, the pseudo-item probabilities can be defined as follows:

15 $$\begin{aligned} p(X_{1vi}=x_{1vi}) =&\sigma _x\left[ p^{(D)}\Bigl (x_{1vi}\,|\,\varvec{s} = (0,1), \eta _{1v}, \alpha _{1i}, {\beta }_{1i}\Bigr ), p^{(I)}\Bigl (x_{1vi} \,|\, \varvec{s} = (0,1), \theta _{v}, \lambda _{i}, \delta _i = \beta _{2i}, {\xi }_{i}\Bigr ) \right] \nonumber \\ p(X_{2vi}=x_{2vi}) =&\hspace{18.49428pt}p^{(D)}\Bigl (x_{2vi}\,|\, \varvec{s} = (0,1), \theta _{v}, \alpha _{2i}, \beta _{2i}\Bigr ) \nonumber \\ p(X_{3vi}=x_{3vi}) =&\sigma _x\left[ p^{(D)}\Bigl (x_{3vi}\,|\, \varvec{s} = (0,1), \eta _{2v}, \alpha _{3i}, {\beta }_{3i}\Bigr ), p^{(D)}\Bigl (x_{3vi}\,|\, \varvec{s} = (0,1), \theta _{v}, \alpha _{4i}, {\beta }_{4i}\Bigr ) \right] \nonumber \\ p(X_{4vi}=x_{4vi}) =&\sigma _x\left[ p^{(D)}\Bigl (x_{4vi}\,|\, \varvec{s} = (0,1), \eta _{2v}, \alpha _{3i}, {\beta }_{5i}\Bigr ), p^{(D)}\Bigl (x_{4vi}\,|\, \varvec{s} = (1,0), \theta _{v}, \alpha _{4i}, {\beta }_{6i}\Bigr ) \right] , \end{aligned}$$

where $p^{(D)}$ denotes the GPCM, $p^{(I)}$ the GGUM, and $\sigma$ the probability-aggregating function. Note that the DI-MIRT parameterization is used in two ways, once for modeling the co-occurrence of a dominance and an ideal point process in the midscale pseudo-item, and once to model two dominance processes in the extreme pseudo-items. This IRTree structure is abbreviated $\text {D}_{\eta _1}\text {I}_\theta$–$\text {D}_\theta$–$\text {D}_{\eta _2}\text {D}_\theta$.

As before, the thresholds of co-occurring response processes within one pseudo-item cannot be separated so that common category intercepts are defined for all pseudo-items. For pseudo-item $X_{1vi}$, the intercept is $\tau _{1i} = \alpha _{1i}\beta _{1i} + \lambda _{i}\xi _{i}$, for pseudo-item $X_{2vi}$ it is $\tau _{2i} = \alpha _{2i}\beta _{2i}$, for pseudo-item $X_{3vi}$ it is $\tau _{3i} = \alpha _{3i}\beta _{3i} + \alpha _{4i}\beta _{4i}$, and for pseudo-item $X_{4vi}$ it is $\tau _{4i} = \alpha _{3i}\beta _{5i} + \alpha _{4i}\beta _{6i}$. Importantly, the item location of the trait-based process of midscale responding $\delta _i$ is not estimated independently, but set equal to the threshold of agreement $\beta _{2i}$. This equality constraint implies that respondents whose trait levels are equal to the agreement difficulty parameter (a) have maximal ambiguity regarding the decision to agree or disagree (see pseudo-item $X_{2vi}$), and (b) have maximal probability for a trait-based selection of the middle category (see pseudo-item $X_{1vi}$). The larger the distance of the respondents’ trait levels to the item difficulty, the more clear-cut the agreement decisions and the less likely midscale responses are.

## Simulation study

A simulation study was conducted to evaluate the parameter recovery and model fit of IRTree pseudo-items of co-occurring processes modeled by the DI-MIRT model. The study was based on the $\text {I}_\theta$–$\text {D}_\eta \text {I}_\theta$ IRTree model described in the section “Response style analysis in ideal point items”, which analyzes ideal point items on a six-point rating scale while incorporating an ERS influence in the intensity sub-decisions. We choose this model for the simulations since all six response categories were influenced by co-occurring dominance and ideal point processes. This model of co-occu-rring response processes was compared to two models of sequential processes, in which the intensity pseudo-items were unidimensional and dependent on only one of the two processes, that is, either ERS-based (trait-ERS model of sequential processes; $\text {I}_\theta$–$\text {D}_\eta$) or trait-based (trait-trait model of sequential processes; $\text {I}_\theta$–$\text {I}_\theta$). We evaluated how the co-occurring model performed when it was the true data-generating model, and when it was over-parameterized and fitted to data generated under the models of sequential processes, which are both nested within it. Further, we evaluated how the models of sequential processes performed when fitted to data generated by the co-occurring model, meaning that one of the two intensity processes was ignored in the analysis.

### Data generation

Item response data were generated for each of the three models, which are described by Eqs. 12 and 13 ($\text {I}_\theta$–$\text {D}_\eta \text {I}_\theta$ model of co-occurring processes), or by special cases with unidimensional pseudo-items ($\text {I}_\theta$–$\text {D}_\eta$ trait-ERS model and $\text {I}_\theta$–$\text {I}_\theta$ trait-trait model of sequential processes). One hundred replications were conducted for each of two sample sizes *N*, set to 500 and 1000, and two questionnaire lengths *I*, set to 10 and 20. The person-specific trait levels $\theta _v$ and ERS levels $\eta _v$ were sampled from independent standard normal distributions. The discrimination parameters $\alpha _{i}$, $\lambda _{1i}$, and $\lambda _{2i}$ were drawn from *LogN*(0, 0.25). The item locations $\delta _i$ were drawn from a uniform distribution $U(-3,3)$. The thresholds of ideal point processes were sampled from the distributions $N(-2.2, 0.2)$, $N(-1.3, 0.2)$, $N(-1, 0.2)$, $N(-0.8, 0.2)$, and $N(-0.2, 0.2)$. The means of these threshold distributions were ordered across ordinal response categories, so that the first two correspond to the thresholds of intense disagreement ($\varvec{\xi }_{3i}$), the third to the threshold of the agreement pseudo-item ($\xi _{1i}$), and the last two to the two thresholds of intense agreement ($\varvec{\xi }_{2i}$). For dominance processes, the thresholds $\varvec{\beta }_{1i}$ and $\varvec{\beta }_{2i}$ were defined as the sum of item-specific locations $\beta _i$, which were generated from $U(-1,1)$, and the category-specific deviations $\zeta _{ik}$, which were drawn from $N(-0.5, 0.2)$ and *N*(0.5, 0.2). Item responses were sampled according to the model-implied probabilities.^3^

### Estimation and analysis

All analyses were conducted in R (R Core Team, 2023). Bayesian parameter estimation was performed since the proposed IRTree models with DI-MIRT parameterization are comparably complex and their estimation would probably not be possible within the frequentist framework. We used the software program Stan (Stan Development Team, 2023) and the R package CmdStanR (Gabry et al., 2023). For each generated data set, the three models ($\text {I}_\theta$–$\text {D}_\eta \text {I}_\theta$, $\text {I}_\theta$–$\text {D}_\eta$, and $\text {I}_\theta$–$\text {I}_\theta$) were fitted. Priors were set as follows: $\alpha \sim Gamma(1.5, 1.5)$, $\lambda \sim Gamma(1.5, 1.5)$, and $\tau \sim N(0,5)$. The parameters $\delta$ were given a hierarchical prior with a *N*(0, 5) hyperprior for the mean and nonnegative *N*(0, 5) for the standard deviation. The distributions of $\theta$ and $\eta$ were set to *N*(0, 1) for identifying the models.

Furthermore, we set initial values for the Markov chain Monte Carlo (MCMC) chains in all models. Firstly, this was important to avoid MCMC chains getting stuck in local maxima. The same applies to the GGUM, for which Roberts et al., (2000) proposed to fit a constrained model first, and to use the estimates of this model as initial values for the full model. We followed this procedure and defined three constrained models (corresponding to the three full models), in which the discrimination parameters ($\alpha$ and $\lambda$) and category intercepts ($\tau$) were set equal across items. Secondly, setting initial values also allowed to specify the orientation of the latent continuum, which otherwise would not be identified. To this end, the initial values of the item locations were set in accordance with one of the two possible scale orientations. Thereby, the chains only explore that part of the posterior distribution that aligns with this parameter solution and do not jump to the alternative parameter set. As suggested by Liu and Wang (2016), the signs of the item locations were treated as known, which is why such parameters were initialized with values 1 or -1, depending on the signs of the respective generated data set (for empirical data, the signs of the item locations are obviously not known, so content knowledge can inform the selection of one of the two possible scale orientations; a slightly different procedure for setting the initial values is then used as described in the “Empiricalapplications”). For the constrained models, one chain with 500 warmup iterations and 500 post-warmup iterations was run to derive approximate estimates for the model parameters. The expected a posteriori (EAP) estimates were then used to create initial values for fitting the full model.

**Table 1:** Recovery of person parameters by MAB

Generation	Analysis	Trait $\theta$		ERS $\eta$	
		*I*10	*I*20	*I*10	*I*20
$\text {I}_\theta$–$\text {D}_\eta \text {I}_\theta$	$\text {I}_\theta$–$\text {D}_\eta \text {I}_\theta$	0.346	0.249	0.395	0.296
	$\text {I}_\theta$–$\text {D}_\eta$	0.496	0.375	0.434	0.352
	$\text {I}_\theta$–$\text {I}_\theta$	0.377	0.286		
$\text {I}_\theta$–$\text {D}_\eta$	$\text {I}_\theta$–$\text {D}_\eta$	0.497	0.377	0.351	0.264
	$\text {I}_\theta$–$\text {D}_\eta \text {I}_\theta$	0.502	0.379	0.352	0.265
$\text {I}_\theta$–$\text {I}_\theta$	$\text {I}_\theta$–$\text {I}_\theta$	0.309	0.221		
	$\text {I}_\theta$–$\text {D}_\eta \text {I}_\theta$	0.309	0.221		

For the full model, four chains with 500 warmup iterations and 1000 post-warmup iterations were run. To ensure model convergence and enough independent posterior samples for the estimation of each parameter, the Gelman–Rubin statistic $\widehat{R}$ and the effective sample size were evaluated (for more information on these diagnostics, see Vehtari et al., 2021). If at least one model parameter had an $\widehat{R}$ value greater than 1.05 or either the bulk or tail effective sample size was smaller than 100, more samples were drawn (in steps of 500, up to 3000 post-warmup iterations). By this procedure, convergence was achieved for all models while keeping the computation time reasonable for models which provided good diagnostic values after fewer samples.^4^

It is important to note that despite the careful choice of initial values and the interim step of fitting a constrained model, by chance, some MCMC chains may move to either a local maximum or to the area of the posterior distribution which corresponds to the solution with inverted scale orientation. This becomes apparent in that the model does not converge or that the signs of estimated item locations are inverted compared to the initial values. Since this only occurs in very few instances, the model can simply be re-fitted with a different seed. In the simulation study, we ensured that all models converged to the solution of the scale orientation corresponding to that of the data generation to ensure sensible results for the parameter recovery.

**Table 2:** Recovery of item parameters by MAB

Gen.	Analysis	$\tau$		$\delta$		$\lambda _1$ (Agree. $\text {I}_\theta$)		$\alpha _1$ (Int. $\text {D}_\eta$)		$\lambda _2$ (Int. $\text {I}_\theta$)	
		*N*500	*N*1000	*N*500	*N*1000	*N*500	*N*1000	*N*500	*N*1000	*N*500	*N*1000
$\text {I}_\theta$–$\text {D}_\eta \text {I}_\theta$	$\text {I}_\theta$–$\text {D}_\eta \text {I}_\theta$	0.320	0.257	0.260	0.209	0.133	0.094	0.108	0.081	0.126	0.095
	$\text {I}_\theta$–$\text {D}_\eta$	1.260	1.230	0.738	0.568	0.188	0.132	0.166	0.163		
	$\text {I}_\theta$–$\text {I}_\theta$	0.505	0.470	0.426	0.353	0.149	0.109			0.252	0.241
$\text {I}_\theta$–$\text {D}_\eta$	$\text {I}_\theta$–$\text {D}_\eta$	0.275	0.206	0.719	0.584	0.192	0.135	0.101	0.070		
	$\text {I}_\theta$–$\text {D}_\eta \text {I}_\theta$	0.286	0.200	0.490	0.412	0.191	0.134	0.104	0.070		
$\text {I}_\theta$–$\text {I}_\theta$	$\text {I}_\theta$–$\text {I}_\theta$	0.290	0.227	0.233	0.184	0.126	0.089			0.113	0.082
	$\text {I}_\theta$–$\text {D}_\eta \text {I}_\theta$	0.300	0.232	0.225	0.180	0.126	0.089			0.119	0.085

The fitted models (i.e., the co-occurring model and the two models of sequential processes) were compared regarding their parameter recovery by mean absolute bias (MAB) of the EAP point estimates. Further, out-of-sample model fit was compared by an approximation of leave-one-out cross-validation based on Pareto smoothed importance sampling (LOO; Vehtari at al., 2017), where small values indicate better fit. The LOO information criterion has been shown to be superior to other commonly used methods of IRT model comparisons such as the AIC or DIC (Fujimoto & Falk, 2023; Luo & Al-Harbi, 2017).^5^

### Results

The comparison of the co-occurring model ($\text {I}_\theta$–$\text {D}_\eta \text {I}_\theta$) with the trait-ERS ($\text {I}_\theta$–$\text {D}_\eta$) and trait-trait ($\text {I}_\theta$–$\text {I}_\theta$) models of sequential processes in terms of recovering person and item parameters is summarized in Tables 1 and 2, respectively. In general, if the co-occurring model was used to generate the data, the model itself provided considerably lower MABs of estimated parameters than both unidimensional models of sequential processes. In contrast, if one of the models of sequential processes was used to generate the data, the co-occurring model yielded MABs of almost equal size. Thus, the co-occurring model successfully adapted to data sets generated by models of sequential processes nested within it, whereas applying such to co-occurring data led to poor parameter recovery.

The evaluation of the out-of-sample model fit (see Table 3) supports these findings: If the data were generated under the co-occurring model, this was superior to the models of sequential processes and was selected as the best-fitting model in all replications. The average LOO values of the models of sequential processes were considerably larger (i.e., indicating worse fit), also when the uncertainty of the LOO estimates is taken into account. In contrast, the differences in the model fit for sequential data were rather small: The respective true model was still selected as the best-fitting model in a large proportion of replications, though in some replications, the co-occurring model provided a better fit. Further, the average LOO values of the co-occurring model were only slightly larger than the values of the respective true model, and such differences were small compared to the standard errors of the LOO estimates. This suggests that the co-occurring model adapted comparably well to the data of sequential processes and successfully captured the restrictions of models nested within it.

Altogether, the simulation study showed that the new DI-MIRT parameterization of IRTree pseudo-items is beneficial for the analysis of item response data and should be preferred over traditional IRTree models of sequential processes. Analyzing data with co-occurring processes under the assumption of sequential processing, that is, ignoring one of two processes, led to poorer model fit and larger errors of estimated parameters. In contrast, there were hardly any negative effects of applying the co-occurring model to data generated by more parsimonious sequential ones. The higher-parameterized co-occurring model entailed greater flexibility and was better able to compensate for possible misspecification.

**Table 3:** Model comparison by LOO

Gen.	Analysis	*N*500, *I*10			*N*500, *I*20			*N*1000, *I*10			*N*1000, *I*20		
		LOO	*SE*	Prop.	LOO	*SE*	Prop.	LOO	*SE*	Prop.	LOO	*SE*	Prop.
$\text {I}_\theta$–$\text {D}_\eta \text {I}_\theta$	$\text {I}_\theta$–$\text {D}_\eta \text {I}_\theta$	14321	99	1.00	27743	144	1.00	28517	139	1.00	54889	205	1.00
	$\text {I}_\theta$–$\text {D}_\eta$	15093	94	0.00	29503	140	0.00	30131	132	0.00	58416	199	0.00
	$\text {I}_\theta$–$\text {I}_\theta$	15304	90	0.00	30134	131	0.00	30502	127	0.00	59561	187	0.00
$\text {I}_\theta$–$\text {D}_\eta$	$\text {I}_\theta$–$\text {D}_\eta$	15146	93	0.95	29597	137	1.00	30158	131	0.98	58812	194	0.99
	$\text {I}_\theta$–$\text {D}_\eta \text {I}_\theta$	15164	93	0.05	29623	138	0.00	30174	132	0.02	58837	194	0.01
$\text {I}_\theta$–$\text {I}_\theta$	$\text {I}_\theta$–$\text {I}_\theta$	14924	91	0.86	29127	134	0.99	29690	128	0.90	58141	188	1.00
	$\text {I}_\theta$–$\text {D}_\eta \text {I}_\theta$	14934	92	0.14	29156	135	0.01	29702	129	0.10	58170	189	0.00

*Note.* LOO = Average LOO value across replications. *SE* = Average standard error of LOO estimate across replications. Prop. = Proportion of replications with smallest LOO

## Empirical applications

To illustrate the benefits of the general IRTree framework with DI-MIRT parameterization under real-world conditions, two empirical applications of co-occurring dominance and ideal point processes are presented. They relate to the two models described in section “IRTree models of co-occurringprocesses”.^6^

### Response style analysis in ideal point items

In the first application example, the co-occurring IRTree model of RS analysis in ideal point items was applied to a data set consisting of item responses of $N=1505$ participants to $I=15$ items measuring attitudes toward sexual practices by a subscale of the National Health and Social Life Survey (Laumann et al., 1992)^7^. The items were rated on a four-point scale, with categories “not at all appealing”, “not appealing”, “somewhat appealing”, and “very appealing”. The ordinal categories $Y_{vi} \in \{0,...,3\}$ were decomposed into two sub-decisions of agreement and intensity as defined in Table 4. With the exception that the intensity pseudo-items had two instead of three options, the co-occurring model as described in the section “Response Style Analysis in IdealPoint Items” was applied.

**Table 4:** Definition of pseudo-items for responses to four-point rating items

Pseudo-item	Ordinal category			
	0	1	2	3
$X_{1vi}$ (Agreement)	0	0	1	1
$X_{2vi}$ (Intensity | agree)	–	–	0	1
$X_{3vi}$ (Intensity | disagree)	1	0	–	–

The data set was previously used as an application example by Jin et al. (2022; using a subset of the data with 1498 respondents), and the authors showed that a trait-ERS model of sequential processes ($\text {I}_\theta$–$\text {D}_\eta$) fitted the data better than an ordinal ideal point model ignoring RS, and than IRTree models under the dominance assumption. Here we analyze the data further and examine whether the co-occurring model ($\text {I}_\theta$–$\text {D}_\eta \text {I}_\theta$) fits the data even better, which would indicate that the decisions about the intensity of responses were additionally influenced by the ideal point trait.

We used the same analysis scheme as described in the simulation study. The initial values for identifying the orientation of the latent continuum were set on the basis of the estimates reported by Jin et al. (2022). To this end, constrained models without setting initial values were fitted each. If the order of estimated item locations was inverted compared to the results of the previous study, the initial values of item locations and substantive trait levels were set as minus one times the estimates of the constrained model (this was the case for the co-occurring model). Otherwise, the estimates of the constrained model were directly used as the initial values (this was the case for the model of sequential processes). Both models converged with $\widehat{R} < 1.05$.

Model comparisons clearly showed that indeed, the new co-occurring model fitted the data well, since the LOO information criterion of this model (LOO = 33726) was substantially smaller than the one of the model of sequential processes (LOO = 36537). Thus, both a trait-based ideal point process and an ERS-based dominance process were involved in the respondents’ decisions regarding the intensity of their responses. This result provides empirical support for multidimensional sub-decisions and underlines the importance of modeling co-occurring processes in IRTree pseudo-items in addition to sequential ones.

**Table 5:** Estimated discrimination parameters of the co-occurring $\text {I}_\theta$–$\text {D}_\eta \text {I}_\theta$ model fitted to empirical data

Parameter	Mean	Min	Max	Correlation	
				$\alpha _{i}$	$\lambda _{2i}$
$\lambda _{1i}$ (Agreement $\text {I}_\theta$)	1.553	0.913	2.756	−0.015	0.548
$\alpha _{i}$ (Intensity $\text {D}_\eta$)	1.338	0.480	2.909		0.383
$\lambda _{2i}$ (Intensity $\text {I}_\theta$)	1.119	0.764	1.517

**Table 6:** Estimated discrimination parameters of the co-occurring $\text {D}_{\eta _1}\text {I}_\theta$–$\text {D}_\theta$–$\text {D}_{\eta _2}\text {D}_\theta$ model fitted to empirical data

Parameter	Identity leadership			Social identification		
	Mean	Min	Max	Mean	Min	Max
$\alpha _{1i}$ (Midscale $\text {D}_{\eta _1}$)	0.936	0.646	1.210	0.694	0.527	0.921
$\lambda _{i}$ (Midscale $\text {I}_\theta$)	1.819	0.691	2.527	1.373	1.178	1.747
$\alpha _{2i}$ (Agreement $\text {D}_\theta$)	3.076	1.739	4.898	1.982	1.074	2.830
$\alpha _{3i}$ (Extreme $\text {D}_{\eta _2}$)	1.642	1.076	2.086	0.879	0.642	1.097
$\alpha _{4i}$ (Extreme $\text {D}_\theta$)	1.351	0.661	2.205	1.398	0.663	2.233

In light of this finding, we further analyzed the discrimination parameters of the co-occurring model (see Table 5), as these provide information about the relative importance of each of the processes for the two sub-decisions. Overall, the estimates of the co-occurring model were consistent with previous studies on co-occurring dominance processes (e.g., Meiser et al., 2019; Merhof & Meiser, 2023): Firstly, the discriminating power of trait-based agreement was larger than that of trait-based intensity judgments, suggesting higher importance of the trait for global agreement compared to fine-grained decisions among agreement or disagreement categories. In addition, trait-based and ERS-based processes appear to have similar impacts on intensity decisions, as indicated by discrimination parameters of comparable size. Moreover, the item-specific discrimination parameters of trait-based agreement correlated positively with those of trait-based intensity, but not with those of ERS-based intensity judgments. This correlation pattern also seems reasonable since decisions made on the basis of one and the same personal characteristic, in this case the substantive trait, can be assumed to be interrelated within a single item, whereas trait-based and ERS-based decisions are considered independent processes. This interpretation is supported by the fact that the person variables (i.e., the trait and ERS factors) were only weakly correlated ($\hat{r}(\theta , \eta ) = -.16$).

### Middle categories in dominance items

The second empirical example of the DI-MIRT parameterization for co-occurring processes relates to modeling middle categories in dominance items. Response time (RT) data were included in the analysis of item responses in order to put the construct validity of estimated IRTree model parameters to the test. To this end, we examined whether RTs were sensitive to the psychological processes reflected by specific parameters of the IRTree model and changed as hypothesized, which would corroborate reasonable substantive interpretations of the IRT estimates and a meaningful model. We used an empirical data set collected by Henninger and Plieninger (2020) and Fladerer et al. (2021), consisting of item responses and corresponding RTs of $N=786$ participants to two questionnaires, the Identity Leadership Inventory ($I=14$) and a scale of Social Identification ($I=6$)^8^. The items were rated on a five-point rating scale, and the ordinal categories were decomposed into three sub-decisions of midscale responding, agreement, and extreme responding as defined in Fig. 6.

In an initial analysis, for which only the item response data were used, the LOO model fit of a co-occurring model described in the section “Middle categories in dominanceitems” ($\text {D}_{\eta _1}\text {I}_\theta$–$\text {D}_\theta$–$\text {D}_{\eta _2}\text {D}_\theta$) was assessed. The model assumes that the sub-decisions of midscale and extreme responding depend on the substantive trait plus the MRS or ERS, respectively. Note that although the items are considered dominance items, the substantive trait is modeled as an ideal point process in the midscale sub-decision, as non-midscale categories are expected to be more likely for respondents whose trait levels are more strongly deviating from the item in either an upward or downward direction. Thus, a dominance and an ideal point process co-occur in the midscale pseudo-item, whereas two dominance processes co-occur in the pseudo-items of extreme responding. In order to test our assumption of trait-based responding being an ideal point process in the midscale pseudo-item, we compared this model with an alternative model in which all processes were considered dominance processes ($\text {D}_{\eta _1}\text {D}_\theta$–$\text {D}_\theta$–$\text {D}_{\eta _2}\text {D}_\theta$). In a second alternative model of sequential processes ($\text {D}_{\eta _1}$–$\text {D}_\theta$–$\text {D}_{\eta _2}$), only the agreement sub-decision was defined as dependent on the trait, so midscale and extreme responding were parameterized by unidimensional models of the respective RS.

All three models converged with $\widehat{R} < 1.05$. Note that even though an ideal point process was modeled in the co-occurring model, it was not necessary to set initial values for identifying the orientation of the latent continuum. This is because the item locations were set equal to the item-specific thresholds of agreement (see Eq. 15), which in turn are inherently identified by the dominance modeling.

The model comparisons revealed that the proposed model of co-occurring processes yielded a considerably better fit (LOO = 30656) than the alternative model with dominance processes (LOO = 31923), demonstrating that trait-based midscale responding was indeed better described by the ideal point rationale. Further, the model also provided a better fit than the model of sequential processes (LOO = 32333), indicating that respondents used both the trait and a RS for the decisions of midscale and extreme responding. The estimated discrimination parameters of the co-occurring model supported this assumption, as they were of substantial size for all processes in all sub-decisions (see Table 6).

A subsequent analysis targeted at the construct validation of the co-occurring model addressed not only the item response data, but additionally the item-level RTs, and both kinds of data were included in a joint model. The item responses were modeled by the co-occurring $\text {D}_{\eta _1}\text {I}_\theta$–$\text {D}_\theta$–$\text {D}_{\eta _2}\text {D}_\theta$ IRTree model. The RTs were log-transformed and analyzed by linear mixed modeling, whereby the predictor variables included IRTree model parameters. Such a joint model allowed to test whether the RTs were sensitive to specific IRTree parameters and changed according to theory-driven hypotheses, which in turn would suggest that the model produced reasonable estimates.

Our hypotheses on how the parameters of the co-occurring IRTree model should affect the RTs were twofold: Firstly, we assumed that the more the item responses were based on the respondents’ individual RS levels, the faster they should be given. The literature suggests that fast responses are associated with low motivation, low data quality, and insufficient effort responding (Callegaro et al., 2009; Bowling et al., 2021; Zhang & Conrad, 2014). Since RS-based responding is a heuristic process requiring less cognitive effort than accurate trait-based responding (Podsakoff et al., 2012; Krosnick, 1991), selecting response categories that match the individual RS should correspond to short RTs. This hypothesis was also investigated by Henninger and Plieninger (2020) in their original work using the data we reanalyzed, and indeed, they found that responses which matched the person-specific RS were given faster. However, they used a two-step approach and obtained estimates of RS levels by an aggregation procedure of dichotomous responses style indicators (i.e., the respondents’ ERS and MRS levels were computed based on the information on whether the given responses were extreme versus non-extreme and midscale versus non-midscale, respectively). Here in contrast, we analyzed the data by the joint model, in which the RS levels were estimated by the co-occurring IRTree model in a one-step approach. Nonetheless, we expected to find similar effects, namely shorter RTs for responses that matched the preferred categories.

Most importantly, our second assumption concerned the ideal point modeling of trait-based midscale responding, and we expected that large distances between the respondents’ trait levels and the items’ locations would result in fast responses. This reasoning relates to a hypothesis that has been frequently described in the literature under terms such as speed-distance or distance-difficulty hypothesis (e.g., Ferrando & Lorenzo-Seva, 2007; McIntyre, 2011; Ulitzsch et al., 2022). It states that a large person-item distance on the latent trait continuum evokes high certainty, which in turn, should be reflected in clear-cut (compared to moderate) responses and shorter RTs.

Part of this hypothesis was also already tested and supported by Henninger and Plieninger (2020), as they found that selecting the middle category was associated with longer RTs, indicating that such responses were related to uncertainty. However, we further analyzed whether RTs were not only dependent on the selected rating category per se (e.g., whether an extreme or midscale category was selected), but additionally affected by the distance of latent person and item locations. We defined the respondents’ locations as the estimated substantive trait levels obtained by the IRTree model and the items’ locations as estimated difficulty parameters of the agreement sub-decision.

The agreement difficulty parameter was used, as it determines for which trait levels the general attitude toward the item statement is rather positive or negative, and thus marks the point of maximal uncertainty. Note that this person-item distance is also part of the IRTree pseudo-item of midscale responding: In this pseudo-item, the substantive trait levels represent the ideal points of the respondents with respect to the midscale sub-decision, and the item locations are set equal to the agreement difficulty parameters (see $X_{1vi}$ in Eq. 15). Therefore, the person-item distance is assumed to affect both the RTs (a higher distance should result in shorter RTs) and the probability of a trait-based selection of middle categories (a higher distance should be associated with a lower probability).

The linear mixed model for predicting the log-transformed RTs of a response *r* given by person *v* to item *i* is defined by

16 $$\begin{aligned} \text {log}(RT_{rvi}) =&\gamma _{000} + \gamma _{1v0} \times X_{1vi} + \gamma _{2v0} \times X_{3vi} + \gamma _{2v0} \times X_{4vi} \nonumber \\&+\gamma _{011} \times |\theta _v - \beta _{2i}| + u_{0v0} + u_{00i} + \epsilon _{rvi} \end{aligned}$$

with

17 $$\begin{aligned} \gamma _{1v0}&= \gamma _{100} + \gamma _{110} \times \eta _{1v}, \nonumber \\ \gamma _{2v0}&= \gamma _{200} + \gamma _{220} \times \eta _{2v}. \end{aligned}$$

The predictors $X_{hvi}$ refer to the IRTree pseudo-items as defined in Fig. 6 and indicate whether a given response was the middle category ($X_{1vi}$) or one of the extreme categories ($X_{3vi}$ or $X_{4vi}$). As those predictors are manifest observations, they do not relate to the IRTree model and were merely included as control variables. In addition, random person and item effects ($u_{0v0}$ and $u_{00i}$, respectively) were included to account for the fact that some respondents are generally faster than others and that some items are faster to respond to than others. Predictors resulting from the IRTree model and referring to the substantial hypotheses were the MRS levels $\eta _{1v}$, the ERS levels $\eta _{2v}$, and the person-item distances $|\theta _v - \beta _{2i}|$. The effect of RS levels matching a given response (hypothesis 1) was captured by $\gamma _{110}$ and $\gamma _{220}$. The effect of the person-item distance (hypothesis 2) was captured by $\gamma _{011}$.

**Table 7:** Estimated coefficients of the linear mixed model predicting log-transformed RTs

Level	Predictor	Coefficient	Estimate	95 %-credible interval
Response	Middle category	$\gamma _{100}$	0.041	[0.016; 0.067]
	Extreme category	$\gamma _{200}$	−0.068	[−0.093; −0.042]
Person x Response	MRS level x middle cat.	$\gamma _{110}$	−0.116	[−0.152; −0.079]
	ERS level x extreme cat.	$\gamma _{220}$	−0.127	[−0.155; −0.098]
Person x Item	Person-item distance	$\gamma _{011}$	−0.086	[−0.105; −0.067]

The results of our analysis using the joint model corroborated both hypotheses (see Table 7): Firstly, we found that heuristic, RS-based responding was related to short RTs. Both for midscale and extreme responding, a match of individual preferences with the selected category reduced the predicted RT in the mixed model (Person x Response level). Further, selecting the middle category was associated with on average longer RTs and extreme responses with shorter RTs (Response level). Thus, the closer the selected category was to the middle of the scale, the more time respondents needed, which indicates that such decisions were related to higher uncertainty. Importantly, a larger person-item distance was associated with shorter RTs in addition to this effect of the selected category (Person x Item level). Therefore, the speed-distance hypothesis was supported not only at the level of manifest response categories, but also at the level of latent locations estimated by the IRTree model. This result is original evidence that the absolute person-item distance, regardless of the direction, influenced both the time it took respondents to choose a category and the probability of selecting the middle category (see estimates of $\lambda _{i}$ in Table 6). The direction of this distance, however, affected the probability to agree or disagree with the item (see estimates of $\alpha _{2i}$ in Table 6).

Altogether, the estimates produced by the co-occurring IRTree model affected the RTs in accordance with our theory-driven hypotheses. This suggests that the new DI-MIRT model appropriately captured the co-occurring response processes, and corroborates the construct validity of the applied IRTree model.

## Conclusion

The present article introduced a general IRTree framework for modeling multidimensional response processes with dominance and ideal point item response functions (IRFs). Such response processes (e.g., responding based on the substantive trait or based on response styles; RS) can be defined to be involved in item responding both sequentially across sub-decisions and as co-occurring processes within sub-decisions. Unlike sequential multidimensionality, which can be implemented using existing IRT modeling (see Jin et al., 2022), co-occurring response processes have previously been limited exclusively to dominance models (von Davier & Khorramdel, 2013 e.g., Alagöz & Meiser, 2023; Jeon & De Boeck, 2016; Meiser et al., 2019; Merhof & Meiser, 2023). Therefore, we developed a new multidimensional IRT model of co-occurring dominance and ideal point processes (DI-MIRT model), with which multiple dominance processes, multiple ideal point processes, as well as a combination of both can be included in IRTree pseudo-items in a consistent way. The proposed DI-MIRT parameterization expands the toolbox of IRTree models and thereby opens up new application areas for this model class. A wide range of theoretical assumptions about the cognitive processing during item responding can be specified within the new general IRTree framework, in which different components can be flexibly combined in the sense of a modular system. Independent choices can be made regarding the decomposition of ordinal responses into sub-decisions, the assignment of response processes to the sub-decisions, and the selection of IRFs for the individual processes. Such components can be freely defined and adapted to the research question and the data at hand.

A simulation study demonstrated that the proposed IRTree framework with DI-MIRT parameterization of pseudo-items accurately captured co-occurring processes and recovered the person and item parameters well. Furthermore, it also showed good parameter recovery in the case of over-parameterization, that is, when applied to data generated under IRTree models in which multiple response processes were only involved sequentially across pseudo-items. In contrast, if one of the co-occurring processes was ignored and a parsimonious IRTree model of sequential processes was falsely applied, larger errors of estimated parameters and poorer model fit resulted. These findings indicate that multidimensional pseudo-items should be preferred over unidimensional ones, wherever this seems reasonable from a theoretical point of view. This recommendation is well in line with previous research on IRTree models with dominance parameterization, in which multidimensional pseudo-items were likewise found to be better suited to capture diverse data situations than unidimensional ones (Merhof et al., 2023). The DI-MIRT model facilitates to include multidimensional pseudo-items for both dominance and ideal point processes and goes beyond previous MIRT models, which were limited to specific kinds of processes (Bolt & Johnson, 2009; Bolt & Newton, 2011; Falk & Cai, 2016; Jin & Wang, 2014; Javaras & Ripley, 2007; Liu & Wang, 2019; Henninger & Meiser, 2020).

Two empirical examples further demonstrated the advantage of the new IRTree parameterization under realistic conditions. In the first example, a co-occurring model was used to analyze the influence of ERS on responding to ideal point items, in which trait-based responding was modeled under the ideal point assumption and ERS-based responding under the dominance assumption. Both kinds of response processes were considered for modeling the sub-decisions of extreme versus non-extreme responding by using the DI-MIRT parameterization, and the results showed that indeed the trait and the ERS co-occurred in such decisions, which is why ignoring one of the two processes led to a substantially worse model fit. The exemplary IRTree model used for analyzing this data set can be easily adapted to other applications of co-occurring trait and RS effects with differently structured trees, different sub-decisions, or other RS. Further extensions are also conceivable with respect to additional influences apart from RS, such as socially desirable responding, which likewise follows the dominance rationale. Thereby, the default assumption in the literature that traits are dominance processes can be challenged and compared to the alternative ideal point assumption, while taking further response processes into account. Such investigations seem promising, as the previous research has shown that even if items were constructed as dominance items, ideal point models may better describe the response behavior of respondents (Drasgow et al., 2010).

The second empirical example of this article made use of the DI-MIRT parameterization for examining the respondents’ use of middle categories. It was shown that the substantive trait as well as the MRS influenced such decisions, and that multidimensional pseudo-items fitted the data better than the unidimensional ones. The additional analysis of response time data further supported the construct validity of the estimated DI-MIRT parameters, as the relation of parameters and response times was in line with the theory-driven hypotheses. Moreover, the model used in this application demonstrated that response processes do not necessarily adhere to fixed IRFs (i.e., are inherently dominance or ideal point processes), but that it may be beneficial to assign different IRFs to one and the same process across IRTree pseudo-items: Although the items were considered dominance items, meaning that trait-based agreement was modeled as dominance process, trait-based midscale responding was defined as an ideal point process. This choice of IRFs reflected our hypothesis that midscale responding was unlikely for both very high and very low trait levels in relation to the item location, which was indeed supported by the data. Such a varying assignment of IRFs could also be useful for other research questions. For instance, one could assume that the respondents first decide on whether the item generally fits their own attitude (i.e., trait-based agreement follows the ideal point rationale), but subsequently respond according to a more-is-better principle (i.e., fine-grained sub-decisions are reflected by the dominance rationale).

Furthermore, co-occurring dominance and ideal point response processes may exist outside self-reported rating data, such as in the field of educational research and ability measurement: For example, the performance in low-stakes assessments might not exclusively be the result of a dominance process with the probability of correct responding being monotonously increasing with higher ability levels. Instead, respondents with very high ability levels may not feel sufficiently challenged and respond with a somewhat lower effort than others, which may result in a lower-than-expected performance. In such cases, a combination of ideal point and dominance IRFs might be appropriate, resulting a steep increase in expected performance from low to high trait levels and a slight decrease for even higher levels. As responding to performance items can usually not be decomposed into different sub-decisions, and as the responses to such items are typically coded as correct or incorrect, the DI-MIRT model could be used as an ordinal or dichotomous model without implementing it within the IRTree framework. A similar dichotomous DI-MIRT model might be suitable for investigating missing responses in performance tests, where a higher-than-expected number of item omissions could likewise occur for respondents who are not sufficiently challenged.

In addition, a DI-MIRT parameterization of IRTree models could be used for modeling missing responses in Likert-type rating data, for instance, as an extension of the missing model introduced by Jeon and De Boeck (2016). The authors proposed an IRTree model in which respondents first decide on whether they wanted to omit the item based on their omission propensity, and then optionally answer the item and chose one of the available categories based on the substantive trait. As a further development to the original model, the omission sub-decision could be given a two-dimensional parameterization of both the omission propensity and the trait. While the omission propensity can be considered a dominance process, the ideal point assumption seems reasonable for the trait-based response process: Mainly respondents who have moderately high trait levels in relation to the item are expected to omit the response, whereas respondents with very high or low trait levels are unlikely to skip the item, as they should have clear-cut opinions. Thus, the ideal point trait could be combined with the dominance omission propensity in the omission sub-decision using the DI-MIRT model, similar to the modeling approach of middle categories in the present article.

Another possible application of the proposed DI-MIRT model (within or outside the IRTree framework) is the co-occurrence of two ideal point response processes. For instance, a bifactor model may reflect the factor structure of a questionnaire with several interrelated sub-scales. If both the general factor and the specific factors are assumed to follow the ideal point rationale, multidimensional modeling of several ideal point processes would be required, which can be achieved by the DI-MIRT model.

A limitation of the proposed DI-MIRT approach is the assumption that the same composition of response processes holds for all respondents.^9^ It seems likely that individuals differ in what response processes they use to what extent in empirical data, especially if the circumstances of the data collection vary (e.g., because the respondents’ motivation or perceived time pressure differ). As a result, some respondents may derive their answers solely based on their substantive trait, while others may additionally use RS. Further, respondents may also differ in how they perceive the item statements and the rating scale, which could lead to some respondents applying trait-based responding in a dominance way, while others may rather respond in an ideal point fashion. The model proposed here cannot account for such heterogeneity between respondents and instead will reveal the average group-level response behavior. More detailed insights about the item response process would be obtained if interindividual differences were considered, for example, by extending the DI-MIRT model by a person mixture. Though such an approach appears very promising from the theoretical perspective, future studies would be needed to evaluate the practical feasibility of estimating group-specific parameters in the DI-MIRT framework.

A further limitation of the present work is that we investigated the co-occurrence of only two response processes at most. We considered this as a plausible assumption within the IRTree framework since more than two processes nevertheless can contribute to item responding across sub-decisions. Moreover, this ensured that the complexity of the models was kept at a reasonable level. Although the DI-MIRT approach comprises a wide range of potentially very complex models, heavily parameterized models including many response processes should be used with caution, as it may become impossible to disentangle and interpret the defined processes. Instead, researchers should specify their models based on theoretical considerations and compare models with increasing complexity against each other in order to select a well-fitting but interpretable model. Thereby, models should be defined in accordance with the research context, substantive question, and data situation. While investigating suitable specifications for certain applications goes beyond the scope of this article, it represents a promising direction for future research. With this in mind, the DI-MIRT model introduced here offers a versatile approach for psychometricians in various fields of research and practice.

## Footnotes

1 The term *sequential* in the context of IRTree models refers to the logical sequence and conditionality of response processes, which does not necessarily imply a temporal sequence.

2 Nevertheless, the assumption of cumulative effects can also be reasonable in ideal point modeling under certain circumstances: For instance, Cui (2008) proposed a multidimensional model for repeated measurements, in which the person-specific latent trait at a given time point *t* was modeled as the sum of the trait at the baseline time point plus the change from baseline to time *t*. Since both of the trait factors are on the same latent scale, their contributions to the response selection can be considered to be additive. In contrast, the present article rather addresses scenarios of multidimensional item responding in which different response processes relate to different constructs, so that their contributions cannot be aggregated in a cumulative way.

3 The R code for generating data sets can be found in the OSF project; https://osf.io/yu4gx/.

4 The Stan model code and an R script illustrating the estimation procedure can be found in the OSF project. The R script also shows how traceplots can be used as an additional check of model convergence.

5 Additional analyses are provided in the OSF project, including recovery by root mean square error and correlation of generated and estimated parameters as well as model fit by the widely applicable information criterion (WAIC; Watanabe, 2010).

6 The Stan code of such models can be found in the OSF project.

7 The data are provided here: https://www.icpsr.umich.edu/web/HMCA/studies/6647

8 The data are made available by the original authors here: https://osf.io/gqb4y/

9 We thank an anonymous reviewer for pointing this out.
